# Distinct Functional Programs in Fetal T and Myeloid Lineages

**DOI:** 10.3389/fimmu.2014.00314

**Published:** 2014-07-07

**Authors:** Elisabeth R. Krow-Lucal, Joseph M. McCune

**Affiliations:** ^1^Division of Experimental Medicine, Department of Medicine, University of California San Francisco, San Francisco, CA, USA

**Keywords:** T cell, myeloid cells, human, immune development, immune function

## Introduction

Pregnancy poses a challenge to normal mechanisms of immune recognition and rejection: both the mother and her fetus are exposed to allogeneic cells from one to the other. In the case of the mother, these cells are fetal cells carrying paternal antigens; in the case of the fetus, they are maternal cells expressing non-inherited maternal alloantigens (1, 2). Since adaptive immune recognition of these alloantigens could result in mutual rejection and an end to the pregnancy, there are extensive mechanisms in place to inhibit such responses, including poor antigen presentation (3), non-canonical MHC expression, and unique placental and decidual immunomodulatory cell populations (4). The reader is referred to several excellent reviews on this subject (4–7).

**Figure 1:**
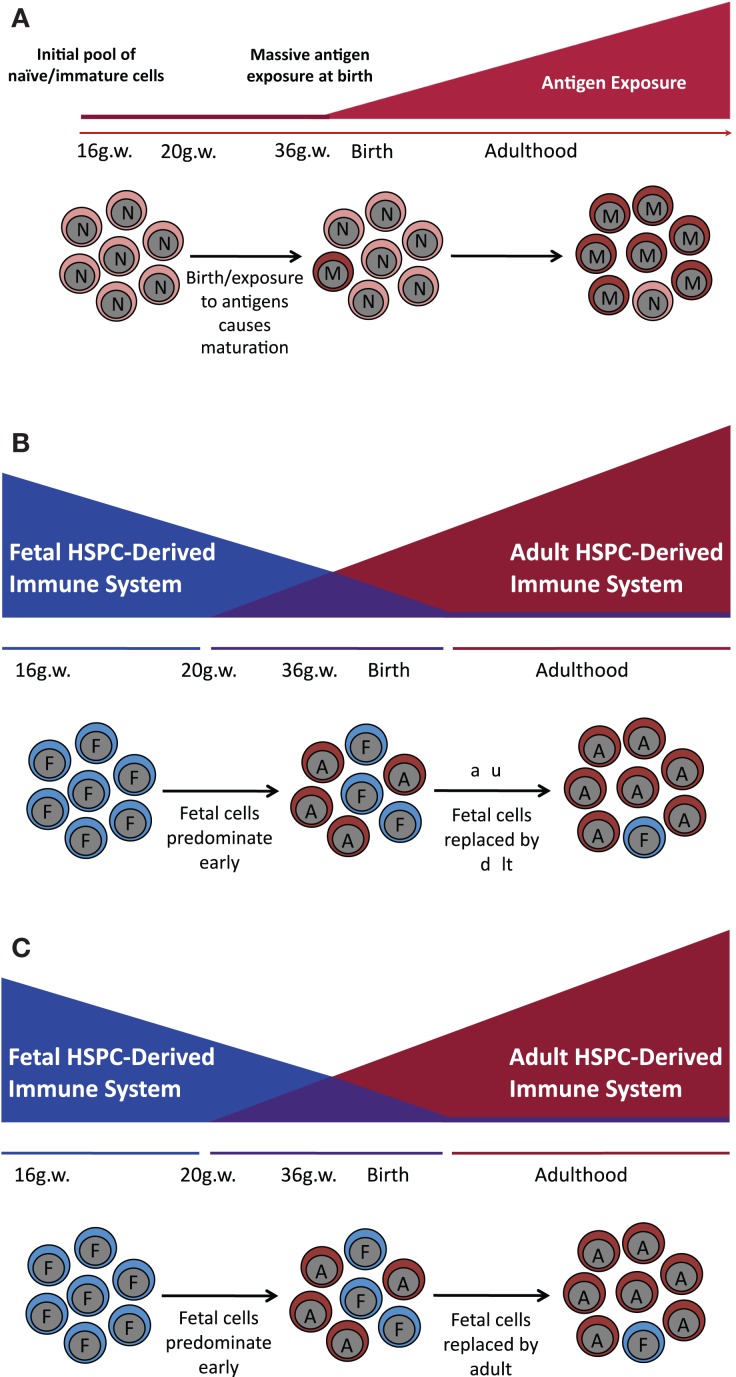
**Models of human immune development**. Throughout different stages of development, fetal T and myeloid cells, as compared to their adult counterparts, specifically populate a subset of tissues including the epithelium (DETC, non-DETC cells, and Langerhans cells), the brain (microglia), and the heart (cardiac macrophages). This differential population of tissues depending on developmental stage suggests that immune maturation does not proceed in a linear fashion and is rather a specifically timed layering of cells with distinct functions, giving rise to specialized tissue-resident populations. **(A)** *Linear model of immune development*. Previous work suggested that the fetal immune system is completely naïve. After birth, it is exposed to massive antigenic doses and matures into the adult immune system. **(B)** *The layered model of immune development*. Several types of HSPC appear sequentially and function at specific times during development in a cell autonomous manner, creating unique layers of HSPC-derived cells with different functional outcomes. During gestation, cells are derived from a fetal HSPC and have a specific functional outcome; while in the adult, the vast majority of the cells come from an adult HSPC that has an adult functional outcome. This model suggests that there is a period between 20 gestational weeks and 6 months to a year after birth where there is an admixture of fetal and adult-derived cells, and that it is this admixture of functionally different cell populations that gives rise to inter-individual differences observed in the neonatal immune response. **(C)** *The maturation model of immune development*. Fetal cells mature into adult cells such that at birth there are cells with a mixture of both fetal and adult characteristics.

Given the inherent difficulties attending experiments in humans, studies of the fetal–maternal interface have focused primarily on inbred strains of laboratory mice. There are, however, major differences between the biology of immune system development of such mice and that found in humans, making it challenging to relate findings in one species to the other. In mice, by example, mature αβ T cells colonize peripheral lymphoid organs during very late gestation and do not fully populate the periphery until after birth (8). By contrast, mature αβ T cells can be found in the periphery of the human fetus as early as 10–12 gestational weeks (5, 9). Thus, early hypotheses posited that *in utero* tolerance was maintained by a passive or inert fetal immune system (similar to that found in the mouse) (Figure 1A). However, current research suggests that there exist distinct fetal programs both in the T and myeloid compartments that contribute to the unique environment *in utero*, both in mice and in humans (Figures 1B,C).

## Fetal T Cell Development and Function

Early work in quail chick embryos demonstrated that thymic T cell development occurs in sequential waves, each of which can be identified by differential stem cell colonization of thymic tissue and by unique TCRs (10). These waves appear to be developmentally regulated as they wax and wane according to embryonic gestational age (10), and further work in mice has identified discrete TCR (γδ) utilization during fetal and neonatal development as compared to the adult TCR (αβ) (11–15). Fetal-derived γδ T cells have limited TCR diversity, suggesting a distinct and limited antigen recognition repertoire (12). Furthermore, these cells appear to localize to specific tissues, including the epithelium (16) and the intestine (17). This localization and restricted TCR repertoire suggest that these fetal-derived cells may play a unique role in barrier sites and, as they are developmentally restricted, may be important for promoting tolerance to skin and gut microbiota in early life.

Because of their distinct TCR repertoire and anatomical location, multiple fetal-derived functional populations have been characterized in mice, including dendritic epidermal T cells (DETCs) and non-DETC γδ T cell populations found in the dermis (18, 19). DETCs are the first T cells and seed the epidermis early in development (20). These cells have been implicated in the inhibition of inflammatory skin conditions (21), protection against cutaneous malignancies (22, 23), and wound repair (24, 25). Non-DETC γδ T cell populations have been shown to be the primary producers of IL-17 (18, 19) in the skin and may play a role in response to infection.

These functions may be indicative of a fetal-specific program, ontologically geared toward appropriate development and maintenance of *in utero* tolerance. Work in humans has demonstrated that while fetal T cells are capable of recognizing and responding to alloantigen *in utero* (1), these cells preferentially differentiate into T regulatory (Treg) cells, capable of suppressing immune responses (1, 26). Furthermore, these studies show that the fetal T cells are derived from a fetal hematopoietic stem/progenitor cell (HSPC) in the fetal liver and fetal bone marrow, which gives rise to downstream progeny that are distinct from those generated by adult bone marrow-resident HSPC.

Taken together, these data suggest that there are developmentally restricted windows of T cell development in which fetal T cells, functionally distinct from their adult counterparts, arise from discrete HSPC, and seed specific anatomical locations (Figure 1B,C).

## Fetal Myeloid Development and Function

The discovery that distinct HSPC give rise to either fetal or adult T cell progeny raises the possibility that there may be differences in other lineages of blood cells as well. Thus, there has been extensive work in human and mouse models elucidating the pathways of regulation of fetal as compared to adult hemoglobin in red blood cells (27–30). Work in mice has meanwhile demonstrated that the presence of fetal-derived B1 cells, with distinct innate-like functions as compared to the adult B2 cells (31–36). Given the multilineage potential of HSPCs, it was also evident that there should be a distinct lineage of fetal- or adult-derived myeloid cells.

In mice, the first hematopoietic progenitors emanate from the extra-embryonic yolk sac and are engaged in primitive hematopoiesis (E7.0–E9.0) (37, 38). “Definitive hematopoiesis” occurs independently in the aorta, gonads, and mesonephros (AGM) region (37, 38). At E10.5, progenitors colonize the fetal liver, the major site of hematopoiesis early in development (38). These waves of hematopoiesis promote egress of various monocyte and macrophage populations, which then give rise to various tissue-resident myeloid populations, including microglia (39), Langerhans cells (LC) (40), and cardiac macrophages (41).

Microglia are the resident macrophage population in the brain and are associated with brain inflammatory diseases. Studies have shown that microglia arise from primitive myeloid progenitors (before E8.0) and are not replaced by circulating monocytes in the adult. Whether or not these fetal-derived cells have distinct functions that differ from an equivalent adult counterpart remains unknown.

Langerhans cells are found in the epidermis of both human and mouse skin. Recent work has demonstrated that LCs are derived from a yolk sac myeloid population during early embryogenesis and then replaced by fetal liver monocytes late in embryogenesis (40). LCs were originally described as pro-inflammatory antigen presenting cells (42); in recent years, however, it has become evident that they can also be involved in – and, indeed, are essential for – the induction of Tregs after infection (43), UV irradiation (44), and glucocorticosteroid stimulation (45). In humans, LCs are able to induce IL-22, but not IL-17, producing T cells (46), potentially suggesting a role in barrier maintenance as opposed to inflammatory processes. LCs also have a limited Toll-like receptor repertoire, including low TLR2, TLR4, and TLR5 expression, leading to attenuated responses to both Gram-positive and Gram-negative bacteria (while leaving viral responses completely intact) (47). Similar to the limited TCR repertoire, this suggests that LCs may be playing a role in tolerization to the skin microbiome.

Recent work highlights the fact that other fetal-derived populations exist in various organs, including lung, liver, spleen, and kidney (41). Furthermore, it has been shown that these populations persist and regenerate *in situ*, rather than being replaced by the circulating adult monocyte pool (40, 41, 48–50). Thus, these functionally distinct fetal-derived myeloid populations persist into adulthood and can affect immunological outcomes throughout the life of the organism.

## Models of Immune Development

Fetal-derived lymphoid and myeloid cells colonize specific anatomical locations and have distinct functions from their adult counterparts. Many of these functions seem tied to barrier integrity and induction of tolerogenic mechanisms. Ontologically, this could be a developmental program designed to allow *in utero* tolerance to non-inherited maternal alloantigens as well as to promote tolerance to commensal bacteria. These distinct functions, as well as the identification of a fetal HSPC (26), suggest that immune maturation in humans may proceed in a layered fashion (51), with a fetal system that pre-dominates *in utero* and an adult system that pre-dominates later in life (Figure 1).

## Conflict of Interest Statement

The authors declare that the research was conducted in the absence of any commercial or financial relationships that could be construed as a potential conflict of interest.
